# Nonpharmacologic treatment for elderly with constipation: a systematic review and meta-analysis

**DOI:** 10.3389/fmed.2025.1644609

**Published:** 2025-09-12

**Authors:** Lingyu Xu, Yan Leng, Peng Dai, Huize Gao, Yunhang Chu, Xingyu Chen, Ming Yang, Xia Li, Tiezheng Yang

**Affiliations:** ^1^College of Traditional Chinese Medicine, Changchun University of Chinese Medicine, Changchun, China; ^2^The Affiliated Hospital of Changchun University of Chinese Medicine, Changchun, China

**Keywords:** non-pharmacologic treatment, elderly, constipation, meta-analysis, efficacy, adverse events

## Abstract

**Objective:**

This study evaluated the efficacy and safety of non-pharmacological interventions such as acupuncture, abdominal massage, ear acupoints, probiotics, and dietary fiber in the treatment of constipation in the elderly.

**Methods:**

Randomized controlled trials (RCTs) published up to March 2025 were retrieved from Cochrane Library, PubMed, Web of Science, Embase, and Chinese databases. The research quality was evaluated using the Cochrane risk of bias assessment tool. Data analysis was performed using RevMan5.4.1 and Stata software. Grade evidence quality was assessed on the analysis’s outcome indicators.

**Results:**

Forty-one studies involving 3,005 patients aged ≥60 years were included. The non-pharmacologic treatment group demonstrated significantly higher efficacy compared to the control group (RR = 1.15, 95% CI = 1.09 to 1.21, *p* < 0.00001), with high heterogeneity (*I*^2^ = 58%, *p* < 0.0001). Subgroup analysis revealed superior therapeutic efficacy of acupuncture (*n* = 15), abdominal massage (*n* = 11), and ear acupoint therapy (*n* = 3) compared to the control group. The incidence of adverse events in the non-drug-treated group was lower than that in the control group (RR = 0.35, 95% CI = 0.16 to 0.74, *p* = 0.006); its heterogeneity was (*I*^2^ = 46%, *p* = 0.04). Meta-analysis of the Constipation-Related Quality of Life Scale (CQLS) revealed that the non-pharmacological treatment group had a more significant therapeutic effect on anxiety or distress. In addition, in the Bristol stool scale, the non-pharmacologic treatment group had better results (standardized mean difference (SMD) = 0.87, 95% CI = 0.14–1.60, *p* = 0.02), and better improvement was achieved after the treatment cycle >4 weeks. In the CSBM scale, the non-pharmacological treatment group showed better efficacy (SMD = 0.44, 95% CI = –0.52–0.12, *p* = 0.03). Symptom score analysis showed that in addition to abdominal distension, eight indicators, including abdominal pain, number of bowel movements, and stool consistency, in the non-pharmacologic treatment group were significantly improved (*p* < 0.05). Some RCTs included in this study had publication bias, and the sensitivity analysis results were robust.

**Conclusion:**

Non-pharmaceutical interventions are better than conventional treatments in the treatment of constipation in the elderly, and long-term intervention has more significant effects. However, due to different intervention regimens, inconsistent treatment time, and methodological defects included in the study, there is a high degree of heterogeneity (CQLS, Bristol, and I^2^ of symptom scores often > 90%). In the future, large-sample, high-quality RCTs are needed to verify their long-term efficacy and related mechanisms.

## 1 Introduction

Constipation, one of the most common gastrointestinal disorders, primarily manifests as dysfunctional bowel movements, reduced bowel movements, and dry and hard feces (1). Epidemiological surveys indicate that the global prevalence of constipation in adults is as high as 15.3%, and it increases with age (2, 3). According to statistics, among patients aged 65 and above admitted to the Geriatric Department, 65% have experienced symptoms and signs of constipation, and 60% have received laxative treatment (4). In addition, in cross-sectional studies conducted in elderly care facilities, 68% of the elderly require regular laxatives (5). Chronic constipation severely impairs the quality of life and mental health of older adults, potentially leading to gastrointestinal and neurological complications, cardiovascular and cerebrovascular events, and even sudden death. It is also associated with colorectal cancer and hepatic encephalopathy, among other conditions (6, 7). Clinical practice guidelines recommend increasing fiber intake (through diet or supplements), increasing water intake in dehydrated patients, and exercise as first-line treatment (8–10). If the above measures fail to significantly improve constipation symptoms, it is recommended to use laxatives such as polyethylene glycol, lactulose, or osmotic laxatives. At present, the use of stimulant laxatives is relatively common, but long-term use may cause a variety of adverse reactions, such as the risk of drug dependence, etc. (11). In addition, there is currently no standardized treatment for constipation, which is particularly prominent in the elderly population, resulting in a large consumption of medical and health resources.

Recently, the advancement of precision medicine has positioned non-pharmaceutical interventions as a prominent focus in clinical research. Multicenter randomized controlled trials and systematic reviews have comprehensively evaluated the evidence-based medical evidence for acupuncture (12), abdominal massage (13), ear acupoints (14), probiotics (15), and dietary fiber (16) in the treatment of constipation. The research results consistently show that the above interventions can significantly improve the symptoms of constipation. Based on the above reasons, we raise two questions: a. Are these non-pharmacologic treatments more effective than lifestyle interventions, placebos, laxatives, and other treatment methods? Are there more adverse reactions? b. When treating various related symptoms of constipation, which symptoms can be effectively improved? Therefore, we conducted a systematic review aimed at evaluating the efficacy and safety of non-pharmaceutical interventions for constipation.

## 2 Methods and materials

### 2.1 Research and design

This study has completed prospective registration for an international systematic review through PROSPERO with registration number CRD420251010371.

### 2.2 Qualification criteria

Our inclusion criteria were as follows: (1) Study subjects were limited to older adults with constipation aged ≥60 years who met the Rome III/IV criteria (17, 18). (2) The research content had to involve the evaluation of the efficacy of non-pharmacological intervention measures. The intervention measures included acupuncture, abdominal massage, ear acupoints, probiotics, and dietary fiber. (3) The main outcome indicators should have included efficacy, adverse events, quality of life scale (CQLS scale), Bristol stool scale, and weekly Complete Spontaneous Bowel Movements (CSBM scale), while the secondary outcome indicators should have included improvement of constipation symptoms after treatment. (4) All the literature types included in this study belong to a randomized controlled experiment (RCT). We followed the following exclusion criteria: (1) Review articles and conference literature were excluded; (2) Clinical trials with subject groups under 60 years of age were excluded; (3) Related studies using drug treatment (including combination of non-pharmaceutical treatment or laxative treatment options) were excluded; (4) Clinical trials with target diseases that might be combined with other concomitant diseases were excluded; (5) Secondary constipation caused by other diseases was excluded. All possible related studies are included in the full-text search scope.

### 2.3 Data selection and extraction

Two authors (Lingyu Xu and Peng Dai) independently extracted data. The extracted content includes study general information (e.g., authors, year), study design, number of participants, intervention measures, treatment duration, and study results, with a structured data extraction form used. Discrepancies in any step were resolved through discussion. In addition, for missing data, we tried to contact authors of the included literature to obtain relevant information. This study aimed to understand efficacy of non-drug treatment for constipation. At present, there are new therapies such as anal lavage, vibration capsules, etc., but this study is limited, and the number of such studies is small and cannot be analyzed. Therefore, we did not include such literature in this study.

We searched English databases (Cochrane Library, PubMed, Web of Science, Embase) and Chinese databases (CNKI, Wanfang, VIP, and CBM). The search period was set to end in March 2025. The keywords used to describe the research population were selected as “constipation,” “dry stool,” “Colonic Inertia,” and “Dyschezia.” These keywords were used in conjunction with keywords of different types of interventions in this system review and RCT-related vocabulary. The search process has no language restrictions. During the literature search process, in addition to database search, references included in the literature are also manually traced, and all documents that meet the standards are imported into the EndNote 21 literature management software for systematic management. See the complete search strategy (Additional Material 1).

### 2.4 Risk of bias assessment

The risk of bias in qualified studies was assessed independently by two review authors (Ling-yu Xu and Peng Dai). The Cochrane Risk of Bias assessment tool (19) is one of the most effective tools for evaluating bias risk in randomized controlled trials (RCTs). As recommended by the Cochrane Handbook, this tool assesses bias risk in six domains: selection bias, implementation bias, measurement bias, follow-up bias, reporting bias, and other biases. The judgment results are classified as “low risk” or “high risk,” and the reason for each judgment will be recorded.

### 2.5 Data analysis

In our meta-analysis, I^2^ and *p*-values were used to evaluate heterogeneity across trials. If I^2^ > 50% or p < *α* (α = 0.1), it indicates that the level of heterogeneity is high; When I ^2^ > 75%, there was a significant difference in the effect size between the studies, and there was high heterogeneity. At this time, we will use the random effect model to summarize the data (20). We will use the Mantel–Haenszel method to express the risk ratio (RR) and 95% confidence interval (CI) for the effects of the interventions included in the study on efficacy and adverse events. As for the impact of interventions on the improvement of constipation symptoms, we will use the standardized mean difference (SMD) to summarize the data; if the median is provided, the mean and standard deviation of the median, range, and sample size are estimated based on the formula proposed by Hozo et al. (21). Finally, we will use RevMan5.4.1 software to summarize the results and generate forest maps, and use the GRADEpro website^1^ to perform GRADE scores for the outcome indicators. At the same time, we used Stata software to conduct a sensitivity analysis on the effect size. This study analyzed publication bias by drawing funnel plots and Egger tests. When the *p* > 0.05, it shows no significant publication bias; if the *p* ≤ 0.05, it indicates that there is publication bias.

## 3 Results

**Figure 1:**
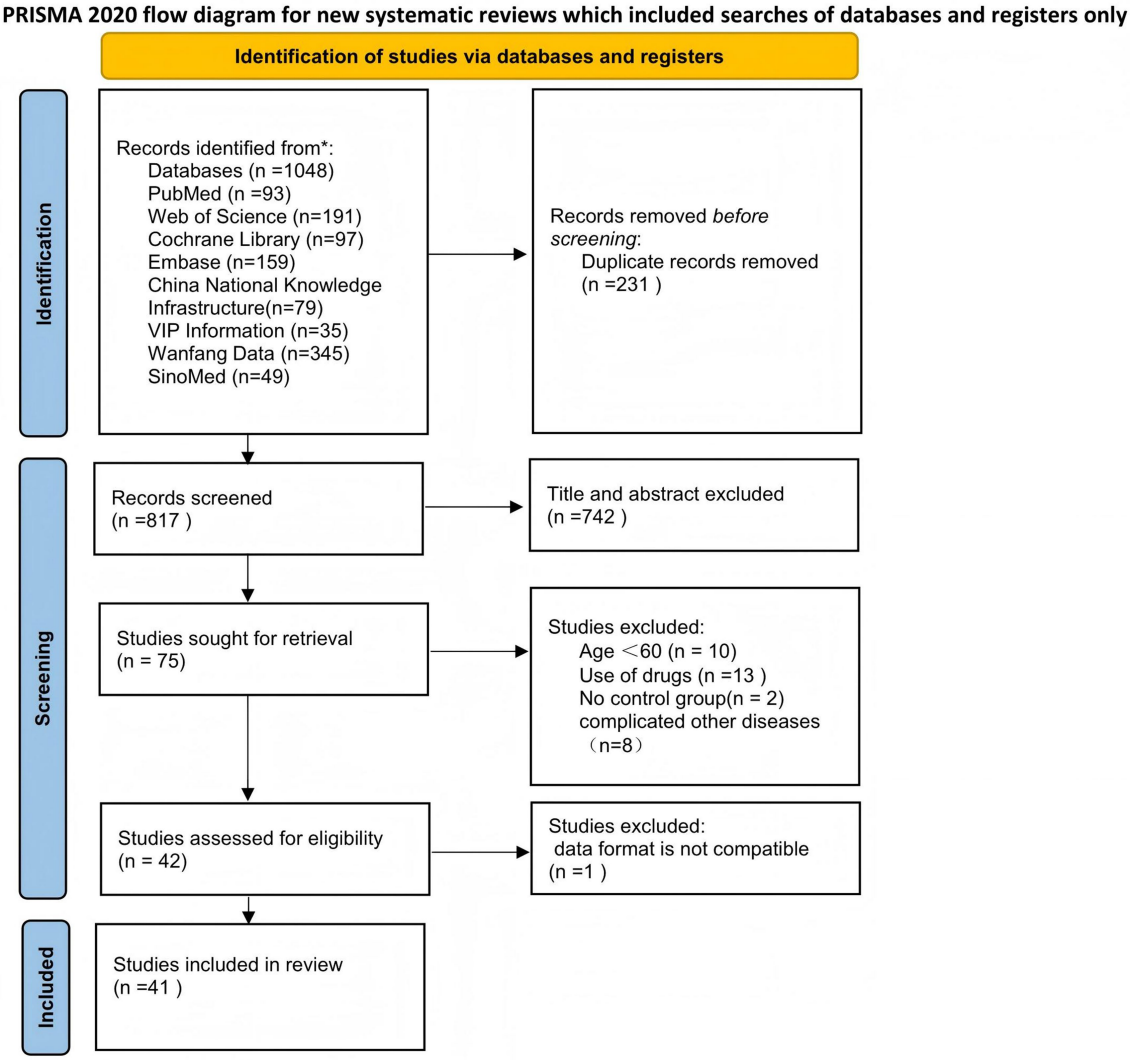
Flow diagram for systematic literature search.

**Figure 2:**
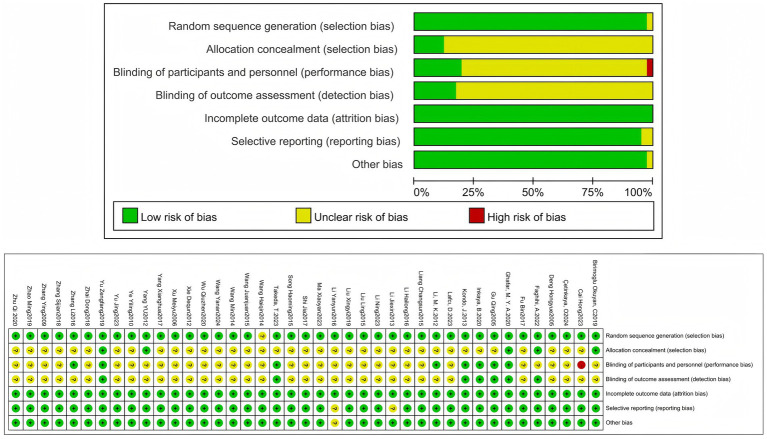
The risk of bias assessment.

According to the systematic search strategy, the total number of initially included literature in this study was 1,048. After deduplication treatment, the efficacy of the literature was reduced to 817. The title and abstract were then initially screened. According to the standards of study design (non-RCT), target population matching degree and intervention measures, 742 studies were screened out, and the remaining 75 randomized controlled trials entered the stage of in-depth evaluation of the full text. After content review, 33 studies that did not meet the inclusion criteria were further excluded. A total of 42 studies that met the inclusion criteria were finally included, but because the data from 1 of them did not include the mean ± standard deviation and could not be converted, they were not included in the subsequent meta-analysis (22). Therefore, the final 41 cases (3,005 elderly people) met the inclusion criteria and were included in the systematic review. The specific search process was found in (Figure 1). The main characteristics of all studies are summarized in Table 1. The overall risk of bias for the included studies is shown in (Figure 2).

**Table 1:** Characteristics of studies included.

Study ID	Random sequence	Sample	Male/Female	Intervention	Frequency	Treatment duration	Outcomes
Birimoglu Okuyan, 2019 (51)	Stratified randomization	35	19/16	Abdominal massage	15 min/d	8 Weeks	1, 3
Çetinkaya, 2024 (57)	Random number table	61	NA	Abdominal massage	15-20 min/d	4 Weeks	4, 5, 6, 7, 8, 9, 10, 12
Ghafar, 2020 (58)	Randomized block	72	40/32	Probiotics	2 times/d	1 Week	2, 12
Lafcı, 2023 (59)	Randomization	48	NA	Abdominal massage	15 min/d	3 Weeks	9, 10, 12
Li, 2012 (60)	Random number table	30	NA	Ear acupoint	NA	3 Weeks	2, 3, 5, 6, 7, 8
Takeda, 2023 (23)	Randomized block	80	44/36	Probiotics	1 time/d	4 Weeks	2, 5, 7, 9, 10, 12, 13
Inkaya, 2020 (24)	Random number table	59	29/30	Abdominal massage	30 min/d	4 Weeks	3, 5, 7
Kondo, 2013 (25)	Minimizing random	66	17/49	Probiotics	1 time/d	16 Weeks	4, 11
Faghihi, 2022 (26)	Random number table	53	27/26	Abdominal massage	20 min/d	2 Weeks	3, 4, 5
Zhang Sijian, 2018 (27)	Randomization	48	20/28	Abdominal massage	20 min/d	1 Week	1
Yu Jing, 2023 (28)	Random number table	60	27/33	Abdominal massage	30 min/d	2 Weeks	1, 4, 5, 12,15
Ye Yiling, 2010(29)	Random number table	60	30/30	Acupuncture	30 min/d	1 Week	1, 2, 5, 14
Xie Dequn, 2012 (30)	Randomization	10	NA	Acupuncture	20-30 min/d	3 Weeks	1
Wang Haiqin, 2014 (31)	Convenience sampling	60	NA	Ear acupoint	10 min/d	5 Weeks	1
Wang Min, 2014 (32)	Random number table	60	34/26	Abdominal massage	30 min/d	3 Weeks	1, 2
Song Haoming, 2015 (33)	Random number table	60	38/22	Ear acupoint	30 min/d	4 Weeks	1, 5, 1
Shi Jia, 2017 (34)	Random number table	60	23/37	Acupuncture	30 min/d	4 Weeks	1, 2, 4, 5
Liu Ling, 2015 (56)	Random number table	100	NA	Acupuncture	20-30 min/d	3 Weeks	1
Li Yanyun, 2016 (35)	Randomization	53	NA	Acupuncture	30 min/d	NA	1
Zhu Qi, 2020 (36)	Random number table	60	25/35	Acupuncture	30 min/d	4 Weeks	1, 3, 5, 12, 15
Li Hailong, 2016 (61)	Randomization	72	35/37	Acupuncture	30 min/d	3 Weeks	1, 12
Wang Juanjuan, 2015 (37)	Random number table	64	35/29	Acupuncture	30 min/d	6 Weeks	4, 5
Li Jiexin, 2013 (38)	Randomization	68	37/31	Acupuncture	20 min/d	3 Weeks	1, 2
Deng Hongyue, 2005 (39)	Randomization	143	66/77	Acupuncture	30 min/d	4 Weeks	1
Gu Qing, 2005 (40)	Completely random	118	48/70	Diet	1 time/d	1 Week	5, 8, 12
Zhai Dong, 2018 (41)	Randomization	52	23/29	Acupuncture	1 time/W	8 Weeks	1, 3, 4, 9, 12, 15
Cai Hong, 2023 (42)	Random number table	60	19/41	Acupuncture	15 min/d	4 Weeks	1, 3, 4
Fu Bin, 2017 (43)	Random number table	108	46/62	Abdominal massage	30 min/d	4 Weeks	1, 2, 8, 9, 13
Li Ning, 2023 (44)	Random number table	80	43/37	Abdominal massage	30-40 min/d	4 Weeks	1, 5, 8, 10, 12, 13
Liang Changsun, 2015 (62)	Random draw	180	NA	Acupuncture	30 min/d	2 Weeks	1, 2, 8, 9, 10, 13
Liu Xingyi, 2019 (45)	Random number table	120	59/61	Acupuncture	10 min/d	2 Weeks	1, 5, 6, 8, 9, 10, 11, 13
Ma Xiaoyan, 2023 (46)	Randomization	60	31/29	Acupuncture	60 min/d	2 Weeks	1, 8, 9, 12
Wang Yanan, 2024 (55)	Randomization	80	43/37	Acupuncture	3time/W	4 Weeks	1, 3, 5, 12, 15
Wu Qiuzhen, 2020 (47)	Randomization	60	31/29	Ear acupoint	20 min/d	2 Weeks	2, 6, 8, 9, 11
Yang Xianghua, 2017 (48)	Randomization	200	112/88	Ear Acupoint	35 min/d	4 Weeks	1, 5, 14
Yang Yu, 2012 (49)	Random number table	60	29/31	Abdominal massage	30 min/d	4 Weeks	1, 5
Yu Zengfang, 2019 (50)	Random number table	62	29/33	Acupuncture	30 min/d	4 Weeks	2, 9, 12., 15
Zhang Li, 2016 (63)	Randomization	60	42/18	Abdominal massage	40 min/d	1 Week	1, 5
Zhang Ying, 2009 (52)	Randomized block	60	29/31	Ear Acupoint	12-20 min/d	8 Weeks	1, 5, 7, 8, 9, 11, 13, 14
Zhao Ming, 2019 (53)	Random number table	60	24/36	Abdominal massage	40 min/d	2 Weeks	1, 5
Xu Meiyu, 2006 (54)	Randomization	98	54/44	Abdominal massage	10-15 min/d	4 Weeks	1, 2

1. Efficacy; 2. Adverse events; 3. CQLS scale; 4. Bristol stool scale; 5. Total symptoms after treatment; 6. Bloating; 7. Pain; 8. Consistency of stool; 9. Level of force; 10. Infinite bowel movement; 11. Frequency of bowel movement; 12. Number of bowel movements; 13. Defecation time; 14. Time of first bowel movement; 15. S CBMs score; NA: Unable to obtain.

### 3.1 Main outcome indicators

#### 3.1.1 Efficacy

**Figure 3:**
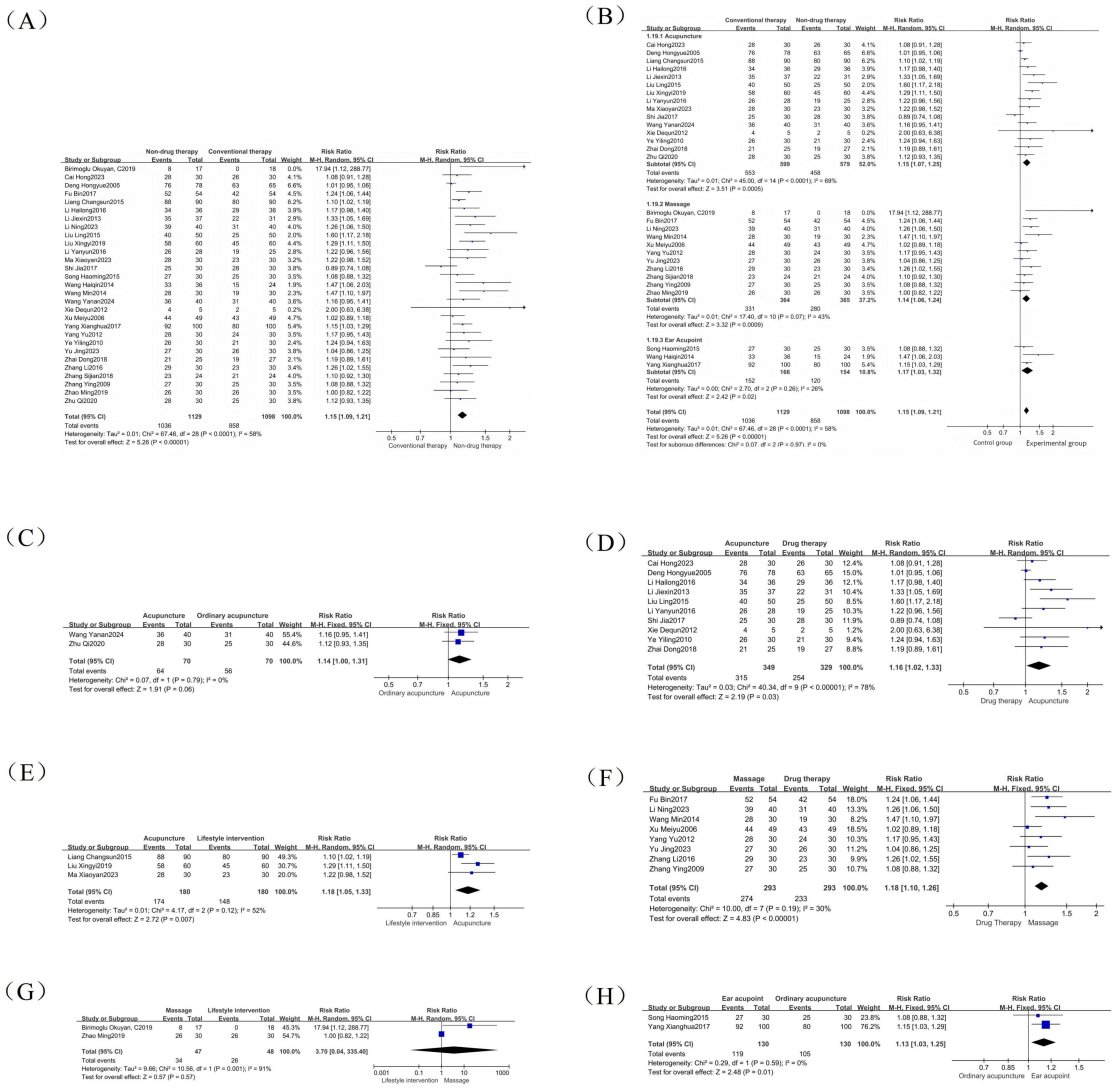
Efficacy forest map. **(A)** Efficacy forest map; **(B)** Efficacy subgroup forest map; **(C)** Common acupuncture treatment forest map in the acupuncture group; **(D)** Drug treatment forest map in the acupuncture group; **(E)** Lifestyle intervention forest map in the acupuncture group; **(F)** Drug treatment forest map in the abdominal massage group; **(G)** Lifestyle intervention forest map in the abdominal massage group; **(H)** Common acupuncture treatment forest map in the ear acupuncture group.

Twenty-nine studies (19, 23–50) were included in the analysis of this indicator, and reported a RR values. The results showed a total of 2,227 participants, with 1,036 (91.76%) in the non-pharmacological treatment group and 858 (78.14%) in the control group achieving an effective response. The efficacy of the non-pharmacologic treatment group was significantly higher than that of the control group (RR = 1.15, 95% CI = 1.09 to 1.21, *p* < 0.00001) (Figure 3A), which was statistically significant. There was heterogeneity between the studies (*I*^2^ = 58%, *p* < 0.0001), so the random effect model was used. The intervention type is used as the basis for grouping subgroups and is divided into three groups: acupuncture, abdominal massage, and ear acupoints. The results showed that there was a significant difference in the subgroup heterogeneity compared with the total heterogeneity: the heterogeneity of the acupuncture group is (*I*^2^ = 69%, *p* < 0.0001), the heterogeneity of the abdominal massage group was (*I*^2^ = 43%, *p* = 0.07), and the heterogeneity of the ear acupoint group is (I^2^ = 26%, *p* = 0.26) (Figure 3B). Therefore, we performed a one-by-one removal method for the acupuncture group and found that after 1 study was eliminated (36), the heterogeneity changed significantly (*I*^2^ = 39%, *p* = 0.07).

We conducted further analysis of the subgroup analysis of different control groups using acupuncture, abdominal massage, and ear acupoints as intervention measures, and observed the efficacy of these non-drug therapies on different controls. The results are as follows: The acupuncture treatment group included 15 studies, with the control group receiving conventional acupuncture, drug therapy, or lifestyle intervention. Among these, conventional acupuncture and warm acupuncture were used as clinical controls. The results showed heterogeneity of *I*^2^ = 0% (*p* = 0.06) in the conventional acupuncture group (Figure 3C), *I*^2^ = 78% (*p* = 0.03) in the drug therapy group (Figure 3D), and *I*^2^ = 52% (*p* = 0.007) in the lifestyle intervention group (Figure 3E). There were 11 studies in the abdominal massage treatment group, and the control intervention methods were drug treatment, lifestyle intervention, and ordinary acupuncture treatment. However, as only one study used conventional acupuncture as the control, further subgroup analysis was not feasible. The results showed that the drug treatment group (*I*^2^ = 30%, *p* < 0.00001) (Figure 3F) and lifestyle intervention group (*I*^2^ = 91%, *p* = 0.57) (Figure 3G). There were 3 RCTs in the ear acupoint treatment group. The intervention methods of the control group were ordinary acupuncture treatment and lifestyle intervention. However, because lifestyle intervention only existed in one of the studies, only the ordinary acupuncture treatment group was analyzed, and the result was (*I*^2^ = 0%, *p* = 0.01) (Figure 3H). Therefore, we speculate that the source of efficacy heterogeneity may be due to differences in intervention methods.

#### 3.1.2 Adverse events

**Figure 4:**
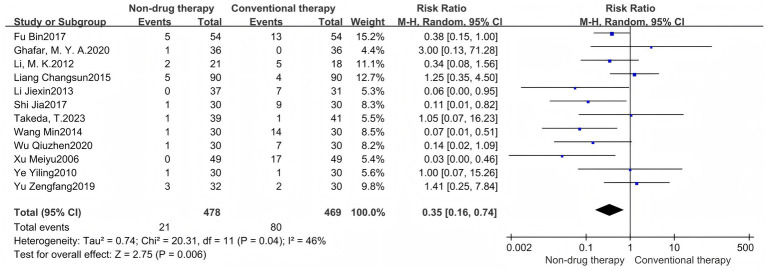
Adverse events forest map.

A meta-analysis of 12-item RCTs was performed to report RR values. The results showed that there were 947 cases in total. The number of adverse events in the non-pharmacologic treatment group was 21 (1.39%), and 80 (17.06%) in the control group. The incidence of adverse events in the non-pharmacologic treatment group was significantly lower than that in the control group (RR = 0.35, 95% CI = 0.16 to 0.74, *p* = 0.006) (Figure 4), which was statistically significant. The heterogeneity between the studies was low (*I*^2^ = 46%, *p* = 0.04), so the random effect model was used.

#### 3.1.3 Constipation quality of life scale

**Figure 5:**
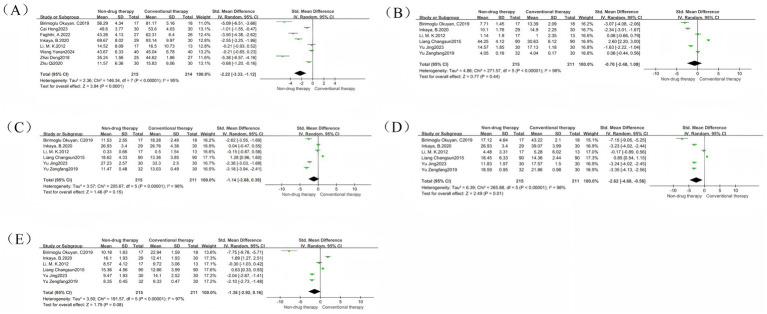
Constipation quality of life scale forest map. **(A)** Constipation quality of life scale forest map; **(B)** Physical discomfort subgroup forest map; **(C)** Psychosocial discomfort subgroup forest picture; **(D)** Anxiety or distress subgroup forest picture; **(E)** Satisfaction subgroup forest picture.

Constipation-Related Quality of Life Scale (CQLS) is a measurement tool for assessing the quality of life of patients with constipation. The tool mainly includes physical discomfort, psychosocial discomfort, anxiety or distress, and satisfaction. It is used to compare and quantify the effects of constipation on the physiological, psychological, and social functions of patients to evaluate the relevant intervention effects (51). A meta-analysis of 8 RCTs was conducted, with a total of 429 participants reported as SMD. The results showed that the CQLS scale in the non-drug-treated group was significantly lower than that in the control group (SMD = –2.22, 95% CI = –3.33 to −1.12, *p* < 0.0001) (Figure 5A), which was statistically significant. There was significant heterogeneity between the studies (*I*^2^ = 95%, *p* < 0.00001), so the random effect model was used. The subscale is used as the basis for grouping of subgroups, and is divided into Physical discomfort, Psychosocial Discomfort, Anxiety or Distress, and Satisfaction.

The Physical discomfort group had 6 RCTs, with a total of 426 participants, and was reported as SMD. Results There was no difference in Physical discomfort scores between the non-pharmaceutical treatment group and the control group (SMD = –0.70, 95% CI = –2.48 to 1.09, *p* = 0.44) (Figure 5B).

The results of the meta-analysis of the Psychosocial Discomfort group showed that there was no difference in the Psychosocial Discomfort score between the non-pharmaceutical treatment group and the control group (SMD = –1.14, 95% CI = –2.68 to 0.39, *p* = 0.15) (Figure 5C).

The results of the meta-analysis of the Anxiety or Distress group showed that the Anxiety or Distress scores in the non-pharmaceutical treatment group were significantly lower than those in the control group (SMD = –2.62, 95% CI = –4.68 to −0.56, *p* = 0.01) (Figure 5D), which was statistically significant.

The results of the meta-analysis of the Satisfaction group showed that there was no difference in Satisfaction scores between the non-pharmaceutical treatment group and the control group (SMD = –1.38, 95% CI = –2.92 to 0.16, *p* = 0.08) (Figure 5E).

#### 3.1.4 Bristol stool scale

**Figure 6:**
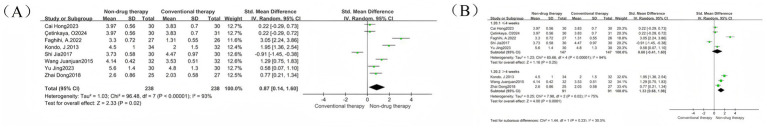
Bristol stool scale forest map. **(A)** Bristol stool scale forest map; **(B)** Bristol stool scale subgroup forest map.

A meta-analysis of 8 RCTs was conducted, with a total of 476 participants reported as SMD. The results showed that the Bristol stool scale in the non-drug-treated group was significantly higher than that in the control group (SMD = 0.87, 95% CI = 0.14–1.60, *p* = 0.02) (Figure 6A), which was statistically significant. There was significant heterogeneity between the studies (*I*^2^ = 93%, *p* < 0.00001), so the random effect model was used. Treatment time was used as the basis for grouping of subgroups and was divided into two groups. The results showed that there were five studies with treatment time within 4 weeks, with a total of 294 participants. There was no difference in Bristol stool scales between the non-pharmacologic treatment group and the control group (SMD = 0.60, 95% CI = –0.41 to 1.60, *p* = 0.25) (Figure 6B); there were three studies with treatment time exceeding 4 weeks, with a total of 182 participants. The Bristol stool scale in the non-pharmacologic treatment group was significantly higher than that in the control group (SMD = 1.33, 95% CI = 0.68 to 1.98, *p* < 0.0001) (Figure 6B), which was statistically significant.

#### 3.1.5 CSBM scale

**Figure 7:**
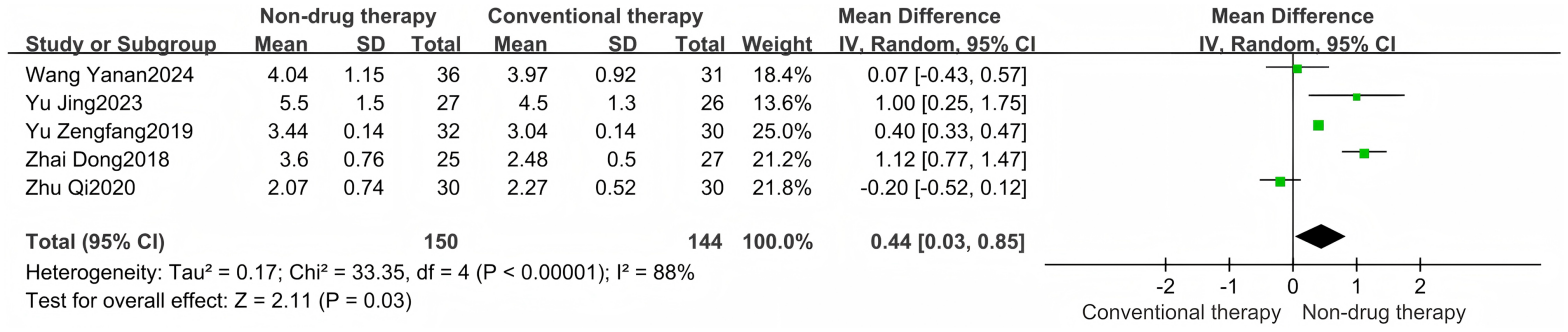
CSBM scale forest map.

A meta-analysis of 5 RCTs involving 294 participants was conducted, with results reported as SMD. The results showed that the CSBM scales in the non-drug-treated group were significantly higher than those in the control group (SMD = 0.44, 95% CI = 0.03–0.85, *p* = 0.03) (Figure 7), which was statistically significant. There was significant heterogeneity before each study (*I*^2^ = 88%, *p* < 0.00001), so the random effect model was used.

### 3.2 Secondary outcome indicators

#### 3.2.1 Symptom points after treatment

**Figure 8:**
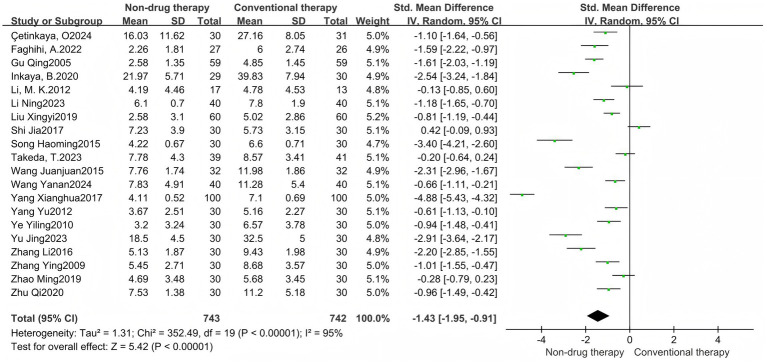
Symptom points after treatment forest map.

A meta-analysis of 20 RCTs consisted of 1,485 participants, including 743 in the non-pharmaceutical group and 742 in the control group, reported with SMD. The results showed that the total score of symptoms after treatment in the non-pharmacologic treatment group was significantly lower than that in the control group (SMD = –1.43, 95% CI = –1.95 to −0.91, *p* < 0.00001) (Figure 8), which was statistically significant. There was significant heterogeneity between the studies (*I*^2^ = 95%, *p* < 0.00001), so the random effect model was used. Symptoms were grouped into subgroups, and are divided into abdominal distension, pain, stool consistency, degree of force, incomplete bowel movement, frequency of bowel movement, number of bowel movements, time of bowel movements, and time of first bowel movements, a total of 9 groups.

**Figure 9:**
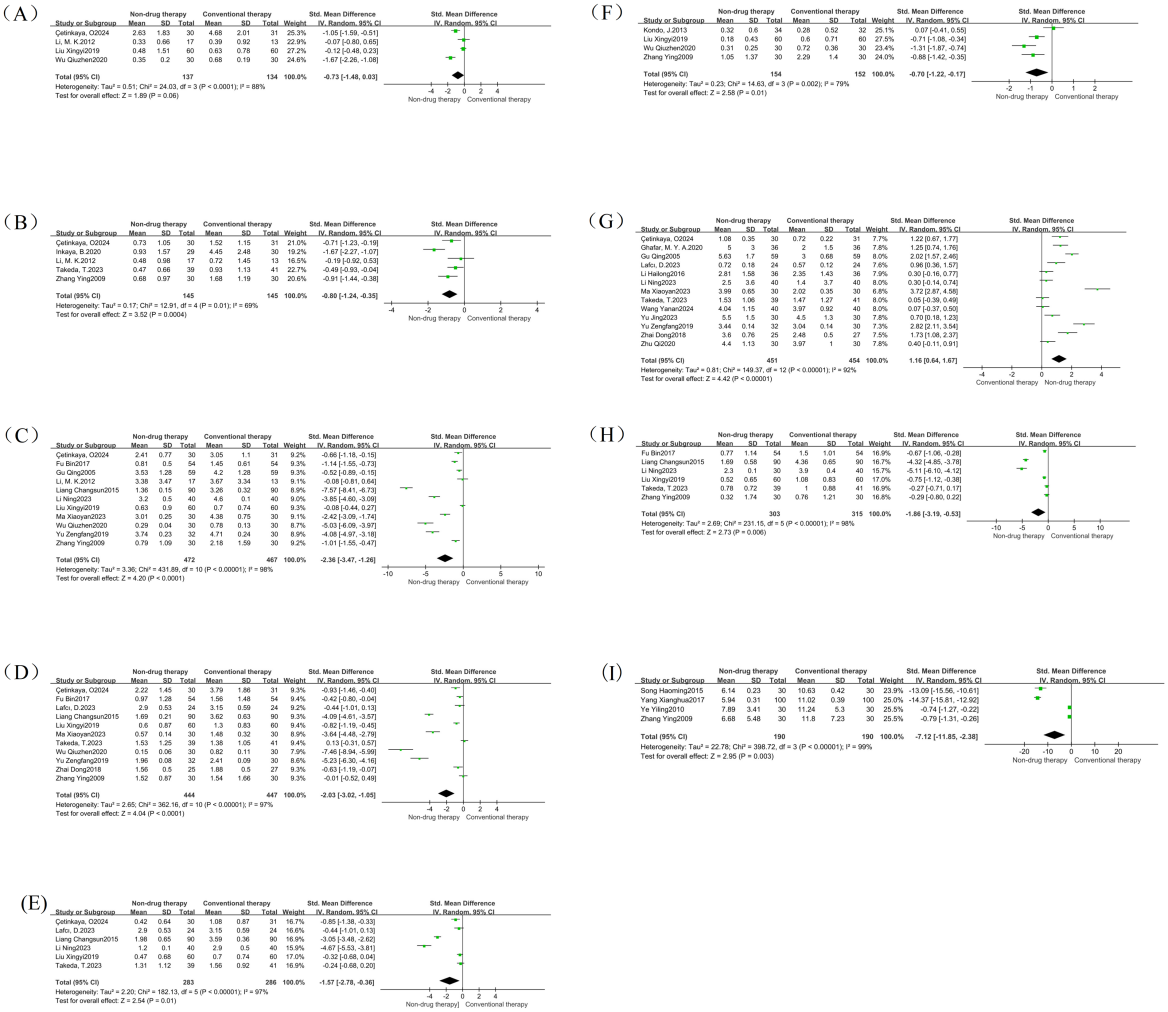
Symptom subgroup forest map. **(A)** Forest map of abdominal distension; **(B)** Forest map of pain; **(C)** Forest map of stool consistency; **(D)** Forest map of degree of force; **(E)** Forest map of incomplete defecation; **(F)** Forest map of frequency of defecation; **(G)** Forest map of number of defecations; **(H)** Forest map of defecation time; **(I)** Forest map of first defecation time.

There were 4 meta-analysis of bloating included in RCTs, with a total of 271 participants reported as SMD. Results There was no difference in abdominal distension scores after treatment between the non-pharmacologic treatment group and the control group (SMD = −0.73, 95% CI = −1.48 to 0.03, *p* = 0.06) (Figure 9A).

A total of 5 RCTs were included in the pain meta-analysis, with a total of 290 participants reported as SMD. Results: The pain score after treatment in the non-pharmacologic treatment group was significantly lower than that in the control group (SMD = –0.80, 95% CI = –1.24 to −0.35, *p* = 0.0004) (Figure 9B), which was statistically significant.

A total of 11 meta-analysis of RCTs were included in the stool consistency, with a total of 939 participants reported as SMD. The results showed that the stool consistency score after treatment in the non-pharmacologic treatment group was significantly lower than that in the control group (SMD = –2.36, 95% CI = –3.47 to −1.26, *p* < 0.0001) (Figure 9C), which was statistically significant.

A total of 11 RCTs were included in the meta-analysis of effort levels, with a total of 891 participants reported as SMD. The results showed that the degree of effort score after treatment in the non-pharmacologic treatment group was significantly lower than that in the control group (SMD = –2.03, 95% CI = –3.02 to −1.05, *p* < 0.0001) (Figure 9D), which was statistically significant.

A total of 6 RCTs were included in the meta-analysis of incomplete bowel movements, with a total of 569 participants reported as SMD. The results showed that the non-pharmacologic treatment group had significantly lower stool scores after treatment than those in the control group (SMD = –1.57, 95% CI = –2.78 to −0.36, *p* = 0.01) (Figure 9E), which was statistically significant.

There were 4 meta-analysis of RCTs included in the defecation frequency, with a total of 306 participants reported as SMD. The results showed that the defecation frequency score after treatment in the non-pharmacologic treatment group was significantly lower than that in the control group (SMD = –0.70, 95% CI = –1.22 to −0.17, *p* = 0.01) (Figure 9F), which was statistically significant.

There were 13 meta-analysis of RCT included in the number of bowel movements, with a total of 905 participants reported as SMD. The results showed that the frequency of bowel movement after treatment in the non-pharmacologic treatment group was significantly higher than that in the control group (SMD = 1.16, 95% CI = 0.64 to 1.67, *p* < 0.00001) (Figure 9G), which was statistically significant.

There were 6 meta-analysis of RCTs included in defecation time, with a total of 618 participants reported as SMD. The results showed that the bowel movement time after treatment in the non-pharmacologic treatment group was significantly lower than that in the control group (SMD = -1.86, 95% CI = –3.19 to −0.53, *p* = 0.006) (Figure 9H), which was statistically significant.

A total of four studies were included in the meta-analysis of first bowel movement time, with a total of 380 participants reported as SMD. The measurement unit is hours, which can directly reflect the therapeutic effect of nonpharmacologic treatment on intestinal peristalsis function, and is an important quantitative index. The results showed that the first bowel movement time after treatment in the non-pharmacologic treatment group was significantly lower than that in the control group (SMD = –7.12, 95%SMD = –11.8 5 to −2.38, *p* = 0.003) (Figure 9I), which was statistically significant.

### 3.3 Post bias

**Figure 10:**
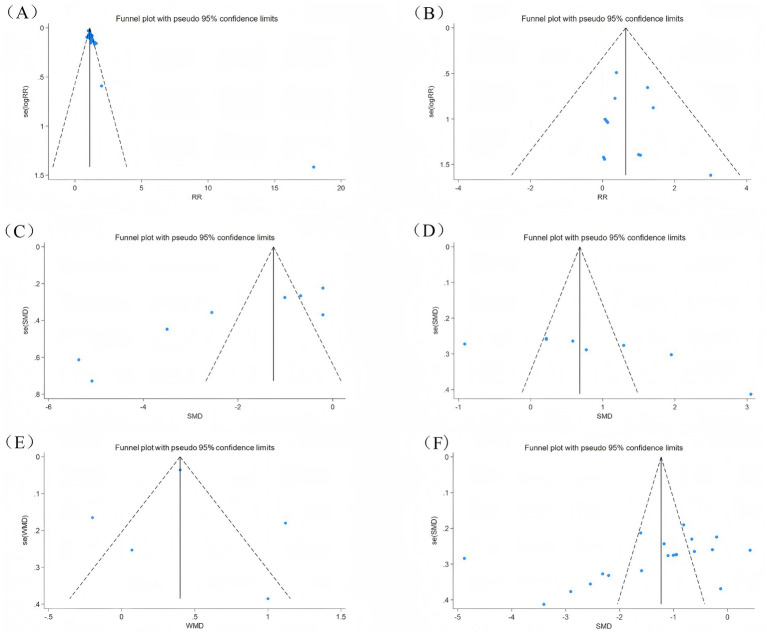
Outcome indicators funnel diagram. **(A)** Efficacy funnel diagram; **(B)** Adverse event funnel diagram; **(C)** CQLS total score funnel diagram; **(D)** Bristol fecal score funnel diagram; **(E)** CSBM scale funnel diagram; **(F)** Symptom score funnel diagram after treatment.

Publication bias was assessed by funnel graph and Egger test on outcome measures (Efficacy, adverse events, CQLS scale, Bristol fecal score, CSBM scale, post-treatment symptom score). Among them, the funnel plots of efficacy, total CQLS scale, Bristol fecal score, and post-treatment symptom score were asymmetrically distributed (Figures 10A,C,D,F), and the Egger test results showed significant publication bias (Table 2). The funnel plots of adverse events and CSBM scales were symmetrically distributed (Figures 10B,E), and the Egger test results suggested that the degree of publication bias was low (*p* = 0.614) (Table 2).

**Table 2:** Post bias.

Numble	Study	*t*	*p*	95% conf. interval	
1	Efficacy	4.31	0.000	1.776769	5.010727
2	Adverse Events	0.52	0.614	−0.9943653	1.599654
3	Constipation Quality of Life Scale	−4.64	0.004	−17.21735	−5.334539
4	Bristol stool scale	2.74	0.034	2.205807	38.59341
5	CSBM Scale	0.13	0.908	−6.344981	6.865464
6	Symptom Points after Treatment	−2.12	0.048	−19.25059	−0.0824376

### 3.4 Sensitivity analysis

**Figure 11:**
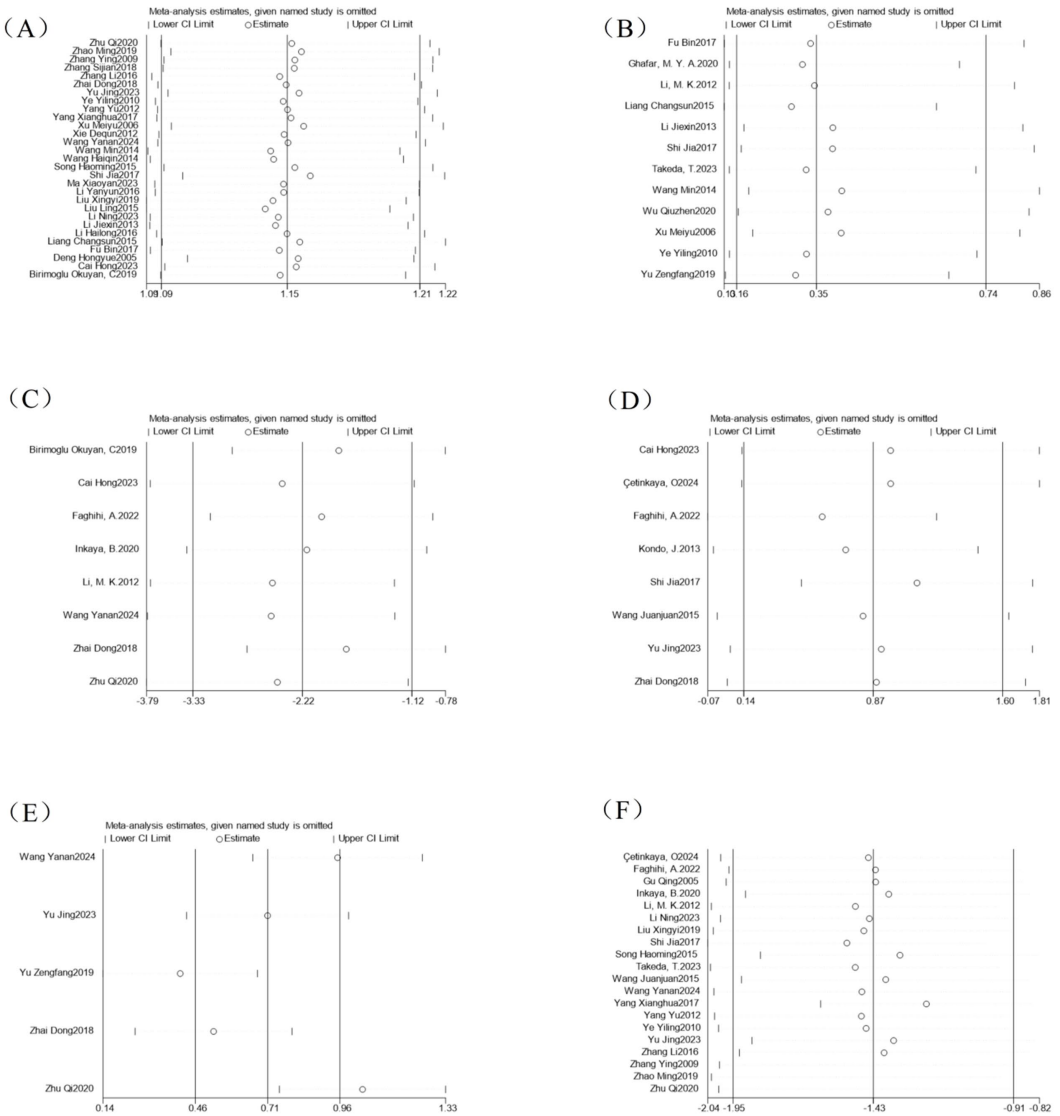
Analysis of sensitivity of outcome indicators. **(A)** Efficacy sensitivity analysis; **(B)** Adverse event sensitivity analysis; **(C)** CQLS scale sensitivity analysis; **(D)** Bristol stool scale sensitivity analysis; **(E)** CSBM scale sensitivity analysis; **(F)** Symptom points after treatment sensitivity analysis.

By excluding individual studies one by one and performing meta-analysis again, there was no significant difference between the pooled effect sizes of each outcome indicator and the entire sample analysis results (Figure 11). Sensitivity analysis showed that excluding any single study did not significantly change the direction and magnitude of the treatment effect.

### 3.5 GRADE scores

We performed a GRADE score on outcome measures in the article to evaluate the rating of evidence for outcome measures in interventions for treating disease. According to (Supplementary File S2), except for the GRADE score of adverse events, the rest are LOW or VERY LOW, which does not mean that our outcome indicators cannot be used to evaluate the degree of disease treatment. Still, it can indicate that the values of these outcome indicators may change under the influence of large samples. However, we can still set the outcome indicators, such as efficacy, CQLS score, and Bristol score, as CRITICAL. In addition, we put the indicators of CQLS-Physical discomfort, CQLS-Psychosocial Discomfort, CQLS-Satisfaction, and Subgroup-Pain as NOT IMPORTANT because we found that the estimated effect range of these indicators was too broad. The effect amount was ineffective, so we determined that the changes in these values could not be used as an outcome indicator for the treatment degree of sexual constipation in elderly patients.

## 4 Discussion

Among the conventional clinical therapeutic drugs, osmotic laxatives such as lactulose and polyethylene glycol, and prokinetic drugs such as mosapride and prucalopride have been confirmed to have clear efficacy in constipation. However, studies have shown (52) that 43% of patients remain concerned about the efficacy and safety of drug treatments. They hope to access alternative treatments, in addition to drug therapy, that alleviate constipation and improve gastrointestinal comfort. Therefore, we carried out a meta-analysis of the efficacy and safety of non-pharmacological therapies.

We included 41 randomized controlled trials involving 3,005 older adults aged ≥60 years, with non-pharmacological interventions including acupuncture, abdominal massage, ear acupoints, probiotics, and dietary fiber, and evaluated their efficacy in the treatment of constipation, CQLS score, Bristol score, CSBM score, total post-treatment symptoms score, and improvement of each symptom. Second, in our meta-analysis of 12 studies that reported adverse events, we found that the non-pharmaceutical treatment group had a lower incidence of such events, and some studies’ follow-up visits showed a better prognosis. The adverse events observed in this study mainly involved local mild discomfort, gastrointestinal symptoms, and a few cases of withdrawal due to discomfort. All of them were recorded and judged by clinical observation, subject self-reporting, and relevant evaluation methods. The overall degree was mild and could be relieved or tolerated by themselves. Then we tested the publication bias of all the literature. Considering that most of the RCT studies are published with positive results, modeling differences, or different regions, the potential impact of publication bias may not be completely ruled out. If more relevant studies are published in the future, the stability of the conclusions of this study can be further verified by correction methods. Sensitivity analysis showed that the analysis results after excluding one study were within the confidence interval, indicating that the results were robust. In addition, some of the studies we included have unclear risk of bias in the fields of allocation concealment and blinding. This is due to the lack of a clear explanation in the original literature, mainly due to methodological limitations, but its impact on the overall conclusion is limited. Regarding the heterogeneity of this analysis, we set this study to exclude combined drug intervention, which may lead to the omission of real-world evidence of a certain mixed treatment strategy, but considering that our topic selection direction is mainly for a single intervention measure, we made such a decision. Secondly, we limit the classification of functional constipation.

Our meta-analysis showed that the non-pharmaceutical treatment group was significantly better than the control group. We used intervention measures as a subgroup grouping method, including acupuncture, abdominal massage, and ear acupoints, to explore whether there were differences in treatment under different intervention methods. The results showed that all were effective, and by observing the total efficacy effect of acupuncture, abdominal massage, and ear acupoint treatment, we believed that the therapeutic effect of acupuncture was slightly better than that of abdominal massage and ear acupoint treatment. When we conducted a subgroup analysis of the acupuncture research, we found that some studies used warm acupuncture and ordinary acupuncture to compare their efficacy, which was better than the intervention of ordinary acupuncture. In addition, the method of burying acupoints has no way to perform further analysis due to the small number of studies, but individual studies have also shown that its efficacy is higher than that of the control group. The intervention methods of different control groups were used as subgroups for secondary analysis. Due to the small number of RCTs of probiotics and dietary fiber, they could not be analyzed separately. The results showed that the acupuncture, abdominal massage, and ear acupoint treatment groups had obvious efficacy advantages compared with the drug treatment control group.

Acupuncture includes manual acupuncture, electroacupuncture, moxibustion, and warm acupuncture. Its therapeutic mechanism primarily involves activating the upper spinal cord, stimulating the vagus nerve, inhibiting the sympathetic nerve, and transmitting peripheral conduction signals to the central nervous system. This regulates the secretion of autonomic nerves and gastrointestinal hormones, thereby promoting colonic smooth muscle contraction and shortening colonic content transit time (53). In addition, acupuncture can also activate the c-kit signaling pathway to promote the proliferation and differentiation of Cajal stromal cells, thereby restoring the electrophysiological activity of the gastrointestinal slow wave rhythm (54), achieving the purpose of improving constipation symptoms. A clinical study by Zengfang Yu (55) showed that compared with the conventional drug control group, acupuncture can significantly increase the number of fully autonomous bowel movements per week in elderly patients with habitual constipation (*p* < 0.05), while effectively alleviating the difficulty of bowel movement, improving CQLS scores, and having good adherence to the patient. In relevant animal experiments, it was also confirmed that acupuncture can regulate autophagy of intestinal glial cells to improve intestinal motor function, and its mechanism may be related to the inhibition of the PI3K/AKT/mTOR signaling pathway (64).

As a non-invasive treatment method, abdominal massage has the advantages of safety, comfort, and good patient tolerance. The mechanism is first based on mechanosensitive neurons in the intestinal ganglion, and through specialized ion channels, it senses mechanical stimulation in the intestinal cavity (65), to trigger the contraction of the intestinal smooth muscle, and generates intestinal peristalsis waves (19) (66). Secondly, the somatic autonomic reflex also plays an important role in abdominal massage therapy. Acting on the connective tissue of the fascial tissue through physical abdominal massage, subcutaneous pressure receptors can be activated to generate afferent nerve impulses and induce autonomic reflexes, thereby enhancing parasympathetic tone (67, 68). In addition, local intestinal deformation stimulation can specifically activate the intestinal wall pull receptors, increase the frequency of the pacing potential of the gastric antrum through the cholinergic pathway, enhance the gastric and colon reflex arc activity, and cause the colon and rectum to produce propulsive peristalsis (69). Çetinkaya’s (20) study found that after 4 weeks of abdominal massage, the severity of constipation in the intervention group decreased from baseline 40.6 ± 10.0 to 16.0 ± 11.6 points, which was statistically significant compared with the treatment group (*p* < 0.05), indicating that abdominal massage has an important therapeutic effect on constipation.

As another non-invasive therapy, ear acupoint compression also shows its unique effect in the treatment of constipation. By placing medicinal beans or magnetic beads on specific ear acupoints, they are given compression and stimulation, causing them to feel acid, numbness, swelling, and pain. Its mechanism is similar to the effect of the vagus nerve to stimulate intestinal smooth muscle contraction and gland secretion (70, 71).

In clinical studies of Bristol scores, we found that treatment cycles were positively correlated with the degree of improvement in fecal traits. When 4 weeks was used as the basis for subgroup division, there was no statistically significant difference in the improvement of Bristol scores between the non-drug-treated group and the conventional control group in the short-term intervention group (treated time ≤4 weeks). It is worth noting that Wang Juanjuan (56)‘s study showed that the Bristol score reached 4.14 ± 0.42 after 4 weeks of intervention in the non-pharmaceutical treatment group, which was significantly higher than that in the control group (*p* < 0.05), and the 4-week follow-up data showed that the long-term efficacy of this regimen had a persistent advantage. This result shows that non-pharmacological treatment plans may require sufficient treatment cycles to fully utilize their clinical efficacy and obtain long-term benefits.

In clinical studies of CQLS scores, the analysis results showed that the non-pharmacologic treatment group had obvious advantages over the control group in terms of overall psychosocial function improvement (*p* < 0.0001). However, after using its subscale as the basis for subgroup grouping, we found that no statistical differences were observed in the therapy in terms of physical symptoms, cognitive function, and satisfaction. This result suggests that non-pharmaceutical intervention has a certain effect on emotional regulation, but the improvement effect in other aspects still needs to be extended treatment time or further verified in combination with other intervention strategies. In addition, in the meta-analysis of the CQLS score, the included studies did not involve placebo treatment. Therefore, it is necessary to design a strict placebo-controlled experiment in future studies to determine which of the real effects and the patient’s psychological effects have a greater impact on emotional regulation. The symptom analysis results showed that, except for the abdominal distension scores, which did not differ significantly compared with the control group, all other symptoms were significantly improved, showing significant advantages of non-drug therapy.

In clinically controlled studies of the CSBM scale, we found that non-pharmacological treatment groups can improve the number of autonomous bowel movements per week in elderly patients, and the mechanism may be related to intestinal dynamics.

Overall, non-pharmacological treatments have shown significant efficacy in the intervention of constipation in the elderly, and have the advantages of safety, high compliance, and good patient acceptance. However, due to the limitations of the enrollment standards of this study, there are fewer RCTs involving probiotics and dietary categories, and they were not discussed further in the subgroup analysis. A key strength of this study is its pioneering analysis of non-pharmacological therapies in elderly populations, confirming their efficacy and safety. In addition, the subgroup analysis also discussed the impact of different intervention methods and treatment time on the treatment of senile constipation. The shortcomings of this study are that some studies have significant heterogeneity, which may be due to the diversity of intervention methods, different treatment cycles, and large sample sizes. Some intervention measures included in the study have a small number of studies and cannot be further subgroup analyzed. It is hoped that more scholars will conduct more RCT studies on non-pharmacologic treatments such as exercise and probiotics, so as to provide more diversified methods for the treatment of senile constipation. The low GRADE scores for some outcomes primarily reflect heterogeneity in study methodologies (such as intervention plan, course of treatment difference) and sample size limitation, which suggests that the conclusion needs to be carefully interpreted in combination with clinical practice. Finally, more attention should be paid to the safety of non-drug therapy in elderly patients. Compared with the side effects of liver and kidney metabolism brought by drugs, non-drug therapy intervention methods are more important for the long-term benefits of patients.

## Data Availability

The datasets presented in this study can be found in online repositories. The names of the repository/repositories and accession number(s) can be found below: All the data in this article are provided in the article.

## Footnotes

^1^ https://gdt.gradepro.org/app/
