# Supplementary material for: Nonpharmacologic treatment for elderly with constipation: a systematic review and meta-analysis
Source: Front Med (Lausanne). 2025 Sep 12;12:1644609. doi: 10.3389/fmed.2025.1644609 (PMC12463846; doi:10.3389/fmed.2025.1644609)
Supplement: Supplementary file 1 [file Data_Sheet_1.PDF]

Embase

#1 'constipation'/exp

#2 'colonic inertia':ti,ab,kw OR 'dyschezia':ti,ab,kw

#3 #1 OR #2

#4 'aged'/exp OR 'aged'

#5 'elderly':ti,ab,kw OR 'olderly':ti,ab,kw

#6 #4 OR #5

#7 'randomized controlled trial':ti,ab,kw

#8 #3 AND #6 AND #7

#9 'acupuncture':ti,ab,kw OR 'acupuncture therapy':ti,ab,kw OR 'electroacupuncture':ti,ab,kw OR 'pharmacopuncture':ti,ab,kw OR 'acupuncture, ear':ti,ab,kw OR 'acupunctures, ear':ti,ab,kw OR 'acupuncture, auricula':ti,ab,kw OR 'acupunctures, auricular':ti,ab,kw OR 'auricular acupunctures':ti,ab,kw OR 'auricular acupuncture':ti,ab,kw OR 'ear acupuncture'

#10 'massage':ti,ab,kw OR 'zone therapy':ti,ab,kw OR 'massage therapy':ti,ab,kw OR 'massage therapies':ti,ab,kw OR 'zone therapies':ti,ab,kw OR 'therapies, zone':ti,ab,kw OR 'therapy, zone':ti,ab,kw OR 'therapies, massage':ti,ab,kw OR 'therapy, massage':ti,ab,kw

#11 'probiotics':ti,ab,kw OR 'probiotic':ti,ab,kw

#12 'diet':ti,ab,kw OR 'diets':ti,ab,kw OR 'meals':ti,ab,kw

#13 'exercise':ti,ab,kw OR 'exercises':ti,ab,kw OR 'exercise, physical':ti,ab,kw OR 'exercise, aerobic':ti,ab,kw OR 'physical activity':ti,ab,kw OR 'activities, physical':ti,ab,kw OR 'activity, physical':ti,ab,kw OR 'physical activities':ti,ab,kw

#14 #9 OR #10 OR #11 OR #12 OR #13

#15 #8 AND #14

Cochrane library

#1 MeSH descriptor: [Constipation] explode all trees

#2 (Colonic Inertia):ti,ab,kw OR (Dyschezia):ti,ab,kw

#3 MeSH descriptor: [Aged] explode all trees

#4 (elderly):ti,ab,kw OR (olderly):ti,ab,kw

#5 #1 OR #2

#6 #3 OR #4

#7 #5 and #6

#8 (randomized controlled trial):ti,ab,kw (Word variations have been searched)

#9 MeSH descriptor: [Acupuncture] explode all trees

#10 (acupuncture therapy):ti,ab,kw OR (electroacupuncture):ti,ab,kw OR (Pharmacopuncture):ti,ab,kw OR (Acupuncture, Ear):ti,ab,kw OR (Acupunctures, Ear):ti,ab,kw OR (Acupuncture, Auricula):ti,ab,kw OR (Acupunctures, Auricular):ti,ab,kw OR (Auricular Acupunctures):ti,ab,kw OR (Auricular Acupuncture):ti,ab,kw OR (Ear Acupuncture):ti,ab,kw

#11 MeSH descriptor: [Massage] explode all trees

#12 (Zone Therapy):ti,ab,kw OR (Massage Therapy):ti,ab,kw OR (Massage Therapies):ti,ab,kw OR (Zone Therapies):ti,ab,kw OR (Therapies,

Zone):ti,ab,kw OR (Therapy, Zone):ti,ab,kw OR (Therapies, Massage):ti,ab,kw OR (Therapy, Massage):ti,ab,kw
#13 MeSH descriptor: [Probiotics] explode all trees
#14 (Probiotic):ti,ab,kw
#15 MeSH descriptor: [Diet] explode all trees
#16 (Diets):ti,ab,kw OR (meals):ti,ab,kw
#17 MeSH descriptor: [Exercise] explode all trees
#18 (Exercises):ti,ab,kw OR (Exercise, Physical):ti,ab,kw OR (Exercise, Aerobic):ti,ab,kw OR (Physical Activity):ti,ab,kw OR (Activities, Physical):ti,ab,kw OR (Activity, Physical):ti,ab,kw OR (Physical Activities):ti,ab,kw
#19 #9 OR #10 OR #11 OR #12 OR #13 OR #14 OR #15 OR #16 OR #17 OR #18
#20 #7 AND #8 AND #19

Pubmed
#1 "Aged"[MeSH Terms]
#2 "elderly"[Title/Abstract] OR "olderly"[Title/Abstract] OR #1
#3 "constipation"[MeSH Terms] OR "Colonic Inertia"[Title/Abstract] OR "Dysch ezia"[Title/Abstract]
#4 #2 AND #3
#5 "randomized controlled trial"[All Fields]
#6 #4 AND #5
#7 ((("Acupuncture"[MeSH Terms] OR "acupuncture therapy"[MeSH Terms] OR "Pharmacopuncture"[Title/Abstract] OR "electroacupuncture"[MeSH Terms] OR "acupuncture, ear"[MeSH Terms]) AND "undefined"[All Fields]) AND (("acupun ctural"[All Fields] OR "Acupuncture"[MeSH Terms] OR "Acupuncture"[All Fiel ds] OR "acupuncture therapy"[MeSH Terms] OR ("Acupuncture"[All Fields] AN D "therapy"[All Fields]) OR "acupuncture therapy"[All Fields] OR "acupuncture s"[All Fields] OR "acupunctured"[All Fields] OR "acupunctures"[All Fields] O R "acupuncturing"[All Fields]) AND "Ear"[Title/Abstract])) OR "acupuncture aur icular"[Title/Abstract] OR (("acupunctural"[All Fields] OR "Acupuncture"[MeSH Terms] OR "Acupuncture"[All Fields] OR "acupuncture therapy"[MeSH Terms] OR ("Acupuncture"[All Fields] AND "therapy"[All Fields]) OR "acupuncture th erapy"[All Fields] OR "acupuncture s"[All Fields] OR "acupunctured"[All Fields] OR "acupunctures"[All Fields] OR "acupuncturing"[All Fields]) AND "Auricula r"[Title/Abstract]) OR "Auricular Acupunctures"[Title/Abstract] OR "Auricular A cupuncture"[Title/Abstract] OR "Ear Acupuncture"[Title/Abstract]
#8 "Massage"[MeSH Terms] OR "Zone Therapy"[Title/Abstract] OR "Massage Therapy"[Title/Abstract] OR "Massage Therapies"[Title/Abstract] OR "Zone Ther apies"[Title/Abstract] OR (("therapeutics"[MeSH Terms] OR "therapeutics"[All Fi elds] OR "Therapies"[All Fields] OR "Therapy"[MeSH Subheading] OR "Thera py"[All Fields] OR "therapy s"[All Fields] OR "therapys"[All Fields]) AND "Z one"[Title/Abstract]) OR "therapy zone"[Title/Abstract] OR "therapies massage"

[Title/Abstract] OR "therapy massage"[Title/Abstract]
#9 "probiotics"[MeSH Terms] OR "Probiotic"[Title/Abstract]
#10 "Exercise"[MeSH Terms] OR "Exercises"[Title/Abstract] OR "exercise physical"[Title/Abstract] OR "exercise aerobic"[Title/Abstract] OR "Physical Activity"[Title/Abstract] OR "activities physical"[Title/Abstract] OR "activity physical"[Title/Abstract] OR "physical activities"[Title/Abstract]
#11 "diet"[MeSH Terms] OR "Diets"[Title/Abstract] OR "meals"[MeSH Terms]
#12 #7 OR #8 OR #9 OR #10 OR #11
#13 #6 AND #12

Web of Science Core Collection
#1 TS=(Constipation OR Colonic Inertia OR Dyschezia)
#2 TS=(Aged OR Elderly OR Olderly)
#3 TS=(randomized controlled trial)
#4 #1 AND #2 AND #3
#5 TS=(Acupuncture OR acupuncture therapy OR electroacupuncture OR Pharmacopuncture OR Acupuncture, Ear OR Acupunctures, Ear OR Acupuncture, Auricula OR Acupunctures, Auricular OR Auricular Acupunctures OR Auricular Acupuncture OR Ear Acupuncture)
#6 TS=(Massage OR Zone Therapy OR Massage Therapy OR Massage Therapies OR Zone Therapies OR Therapies, Zone OR Therapy, Zone OR Therapies, Massage OR Therapy, Massage)
#7 TS=(Probiotics OR Probiotic)
#8 TS=(Diet OR Diets OR meals)
#9 TS=(exercise OR Exercises OR Exercise, Physical OR Exercise, Aerobic OR Physical Activity OR Activities, Physical OR Activity, Physical OR Physical Activities)
#10 #5 OR #6 OR #7 OR #8 OR #9
#11 #4 AND #10

知网：
检索以高级检索，主题为”老年人+老年人群”AND”便秘”AND”推拿+推拿疗法”AND”临床研究”
”老年人+老年人群”AND”便秘”AND”膳食+膳食纤维”AND”临床研究”
”老年人+老年人群”AND”便秘”AND”针灸+针灸疗法”AND”临床研究”
”老年人+老年人群”AND”便秘”AND”穴位+穴位贴敷+穴位治疗+针刺穴位”AND”临床研究”
”老年人+老年人群”AND”便秘”AND”益生菌+益生菌治疗”AND”临床研究”
”老年人+老年人群”AND”便秘”AND”益生元”AND”临床研究”
”老年人+老年人群”AND”便秘”AND”耳穴+耳穴疗法”AND”临床研究”
”老年人+老年人群”AND”便秘”AND”运动”AND”临床研究”

维普：
检索以高级检索，主题为
“老年”AND”便秘+大便干燥”AND”按摩+按摩疗法+推拿治疗+推拿疗法+推拿”AND”随机对照试验+随机对照实验+随机对照研究”
“老年”AND”便秘+大便干燥”AND”膳食”AND”随机对照试验+随机对照实验+随机对照研究”
“老年”AND”便秘+大便干燥”AND”针灸+温针灸+电针”AND”随机对照试验+随机对照实验+随机对照研究”
“老年”AND”便秘+大便干燥”AND”穴位+人体穴位+腧穴+穴道”AND”随机对照试验+随机对照实验+随机对照研究”
“老年”AND”便秘+大便干燥”AND”耳穴+耳压+耳埋+耳针”AND”随机对照试验+随机对照实验+随机对照研究”
“老年”AND”便秘+大便干燥”AND”益生菌”AND”随机对照试验+随机对照实验+随机对照研究”
“老年”AND”便秘+大便干燥”AND”益生元”AND”随机对照试验+随机对照实验+随机对照研究”
“老年”AND”便秘+大便干燥”AND”运动”AND”随机对照试验+随机对照实验+随机对照研究”

万方：
检索以高级检索，点击期刊论文、学位论文，主题为
“老年”AND”便秘 or 大便干燥”AND”按摩 or 按摩疗法 or 推拿治疗 or 推拿疗法 or 推拿”AND”随机对照试验 or 随机对照实验 or 随机对照研究”
“老年”AND”便秘 or 大便干燥”AND”膳食”AND”随机对照试验 or 随机对照实验 or 随机对照研究”
“老年”AND”便秘 or 大便干燥”AND”针灸 or 温针灸 or 电针”AND”随机对照试验 or 随机对照实验 or 随机对照研究”
“老年”AND”便秘 or 大便干燥”AND”穴位 or 人体穴位 or 腧穴 or 穴道”AND”随机对照试验 or 随机对照实验 or 随机对照研究”
“老年”AND”便秘 or 大便干燥”AND”耳穴 or 耳压 or 耳埋 or 耳针”AND”随机对照试验 or 随机对照实验 or 随机对照研究”
“老年”AND”便秘 or 大便干燥”AND”益生菌”AND”随机对照试验 or 随机对照实验 or 随机对照研究”
“老年”AND”便秘 or 大便干燥”AND”益生元”AND”随机对照试验 or 随机对照实验 or 随机对照研究”
“老年”AND”便秘 or 大便干燥”AND”运动”AND”随机对照试验 or 随机对照实验 or 随机对照研究”

SinoMed：

检索以高级检索，

#1 "老年"[常用字段:智能] OR "老年人"[常用字段:智能] OR "老年人群"[常用字段:智能]

#2 "便秘"[常用字段:智能] OR "大便干燥"[常用字段:智能]

#3 "随机对照试验"[常用字段:智能] OR "随机对照实验"[常用字段:智能] OR "随机对照研究"[常用字段:智能]

#4 #1 AND #2 AND #3

#5 "按摩"[常用字段:智能] OR "按摩疗法"[常用字段:智能] OR "推拿治疗"[常用字段:智能] OR "推拿疗法"[常用字段:智能] OR "推拿"[常用字段:智能]

#6 "膳食"[常用字段:智能] OR "饮食"[常用字段:智能]

#7 "针灸"[常用字段:智能] OR "温针灸"[常用字段:智能] OR "电针"[常用字段:智能]

#8 "穴位"[常用字段:智能] OR "人体穴位"[常用字段:智能] OR "腧穴"[常用字段:智能] OR "穴道"[常用字段:智能]

#9 "耳穴"[常用字段:智能] OR "耳压"[常用字段:智能] OR "耳埋"[常用字段:智能] OR "耳针"[常用字段:智能]

#10 "益生菌"[常用字段:智能] OR "益生元"[常用字段:智能]

#11 "运动"[常用字段:智能]

#12 #5 OR #6 OR #7 OR #8 OR #9 OR #10 OR #11

#13 #4 AND #12
