# Supplementary material for: Nonpharmacologic treatment for elderly with constipation: a systematic review and meta-analysis
Source: Front Med (Lausanne). 2025 Sep 12;12:1644609. doi: 10.3389/fmed.2025.1644609 (PMC12463846; doi:10.3389/fmed.2025.1644609)
Supplement: Supplementary file 2 [file Data_Sheet_2.PDF]

Author(s): Ling yu Xu;Peng Dai
Question: Nonpharmacologic Treatment compared to Contral for Elderly Constipation
Setting:
Bibliography:

Certainty assessment							№ of patients		Effect		Certainty	Importance
№ of studies	Study design	Risk of bias	Inconsistency	Indirectness	Imprecision	Other considerations	Nonpharmacologic Treatment	Contral	Relative (95% CI)	Absolute (95% CI)		
**Effectiveness**												
29	randomised trials	serious[a]	serious[b]	not serious	not serious	publication bias strongly suspected[c]	1036/1129 (91.8%)	858/1098 (78.1%)	**RR 1.15** (1.09 to 1.21)	**117 more per 1,000** (from 70 more to 164 more)	⨁◯◯◯ Very low[a,b,c]	CRITICAL
**Adverse Events**												
12	randomised trials	serious[a]	not serious	not serious	not serious	strong association	21/478 (4.4%)	80/469 (17.1%)	**RR 0.35** (0.16 to 0.74)	**111 fewer per 1,000** (from 143 fewer to 44 fewer)	⨁⨁⨁⨁ High[a]	CRITICAL
**Constipation Quality of Life Scale**												
8	randomised trials	serious[a]	serious[b]	not serious	not serious	publication bias strongly suspected[c]	215	214	-	SMD **2.22 lower** (3.33 lower to 1.12 lower)	⨁◯◯◯ Very low[a,b,c]	CRITICAL
**CQLS- Physical discomfort**												
6	randomised trials	serious[a]	serious[b]	not serious	serious[d]	publication bias strongly suspected[c]	215	211	-	SMD **0.7 lower** (2.48 lower to 1.09 higher)	⨁◯◯◯ Very low[a,b,c,d]	NOT IMPORTANT
**CQLS-Psychosocial Discomfort**												
6	randomised trials	serious[a]	serious[b]	not serious	serious[d]	publication bias strongly suspected[c]	215	211	-	SMD **1.14 lower** (2.68 lower to 0.39 higher)	⨁◯◯◯ Very low[a,b,c,d]	NOT IMPORTANT
**CQLS- Anxiety/Distress**												
6	randomised trials	serious[a]	serious[b]	not serious	not serious	publication bias strongly suspected[c]	215	211	-	SMD **2.62 lower** (4.68 lower to 0.56 lower)	⨁◯◯◯ Very low[a,b,c]	IMPORTANT
**CQLS- Satisfaction**												
6	randomised trials	serious[a]	serious[b]	not serious	serious[d]	publication bias strongly suspected[c]	215	211	-	SMD **1.38 lower** (2.92 lower to 0.16 higher)	⨁◯◯◯ Very low[a,b,c,d]	NOT IMPORTANT
**Bristol Scale**												
8	randomised trials	serious[a]	serious[b]	not serious	not serious	publication bias strongly suspected[c]	238	238	-	SMD **0.87 higher** (0.14 higher to 1.6 higher)	⨁◯◯◯ Very low[a,b,c]	CRITICAL
**Symptom Points after Treatment**												
20	randomised trials	serious[a]	serious[b]	not serious	not serious	publication bias strongly suspected[c]	743	742	-	SMD **1.43 lower** (1.95 lower to 0.91 lower)	⨁◯◯◯ Very low[a,b,c]	IMPORTANT
**Subgroup-Pain**												
5	randomised trials	serious[a]	serious[b]	not serious	serious[d]	publication bias strongly suspected[c]	145	145	-	SMD **0.8 lower** (1.24 lower to 0.35 lower)	⨁◯◯◯ Very low[a,b,c,d]	NOT IMPORTANT
**Subgroup-Frequency of Defecation**												

4	randomised trials	serious[a]	serious[b]	not serious	not serious	publication bias strongly suspected[c]	154	152	-	SMD **0.7 lower** (1.22 lower to 0.17 lower)	⊕◯◯◯ Very low[a,b,c]	IMPORTANT
**Subgroup-Abdominal Distension**												
4	randomised trials	serious[a]	serious[b]	not serious	not serious	publication bias strongly suspected[c]	137	134	-	SMD **0.73 lower** (1.48 lower to 0.03 higher)	⊕◯◯◯ Very low[a,b,c]	IMPORTANT
**Subgroup-Stool Consistency**												
11	randomised trials	serious[a]	serious[b]	not serious	not serious	publication bias strongly suspected[c]	472	467	-	SMD **2.36 lower** (3.47 lower to 1.26 lower)	⊕◯◯◯ Very low[a,b,c]	IMPORTANT
**Subgroup-Degree of Force**												
11	randomised trials	serious[a]	serious[b]	not serious	not serious	publication bias strongly suspected[c]	444	447	-	SMD **2.03 lower** (3.02 lower to 1.05 lower)	⊕◯◯◯ Very low[a,b,c]	IMPORTANT
**Subgroup-Incomplete Defecation**												
6	randomised trials	serious[a]	serious[b]	not serious	not serious	publication bias strongly suspected[c]	283	286	-	SMD **1.57 lower** (2.78 lower to 0.36 lower)	⊕◯◯◯ Very low[a,b,c]	IMPORTANT
**Subgroup-Number of Defecation**												
13	randomised trials	serious[a]	serious[b]	not serious	not serious	publication bias strongly suspected[c]	451	454	-	SMD **1.16 higher** (0.64 higher to 1.67 higher)	⊕◯◯◯ Very low[a,b,c]	IMPORTANT
**Subgroup-Time of Defecation**												
6	randomised trials	serious[a]	serious[b]	not serious	not serious	publication bias strongly suspected[c]	303	315	-	SMD **1.86 lower** (3.19 lower to 0.53 lower)	⊕◯◯◯ Very low[a,b,c]	IMPORTANT
**Subgroup-Time of First Defecation**												
4	randomised trials	serious[a]	serious[b]	not serious	not serious	publication bias strongly suspected[c]	190	190	-	SMD **7.12 lower** (11.85 lower to 2.38 lower)	⊕◯◯◯ Very low[a,b,c]	IMPORTANT
**Effectiveness-Subgroup - Acupuncture**												
15	randomised trials	serious[a]	serious[b]	not serious	not serious	publication bias strongly suspected[c]	553/599 (92.3%)	458/579 (79.1%)	**RR 1.15** (1.07 to 1.25)	**119 more per 1,000** (from 55 more to 198 more)	⊕◯◯◯ Very low[a,b,c]	IMPORTANT
**Effectiveness-Subgroup - Massage**												
11	randomised trials	serious[a]	not serious	not serious	not serious	publication bias strongly suspected[c]	331/364 (90.9%)	280/365 (76.7%)	**RR 1.14** (1.06 to 1.24)	**107 more per 1,000** (from 46 more to 184 more)	⊕⊕◯◯ Low[a,c]	IMPORTANT
**Effectiveness-Subgroup - Ear Acupoint**												
3	randomised trials	serious[a]	not serious	not serious	not serious	publication bias strongly suspected[c]	152/166 (91.6%)	120/154 (77.9%)	**RR 1.17** (1.03 to 1.32)	**132 more per 1,000** (from 23 more to 249 more)	⊕⊕◯◯ Low[a,c]	IMPORTANT

CI: confidence interval; **RR:** risk ratio; **SMD:** standardised mean difference

Explanations

a. There were large biases in randomization, allocation concealment and blinding in the included studies.
b. Great heterogeneity
c. Funnel plot indicated publication bias.
d. The sample size of the included studies was too small and the confidence interval was wide.
